# Draft genome sequence of *Janthinobacterium lividum* strain MTR reveals its mechanism of capnophilic behavior

**DOI:** 10.1186/s40793-015-0104-z

**Published:** 2015-11-24

**Authors:** Natalia Valdes, Paola Soto, Luis Cottet, Paula Alarcon, Alex Gonzalez, Antonio Castillo, Gino Corsini, Mario Tello

**Affiliations:** Unidad de Apoyo Bioinformático, Departamento de Biología, Facultad de Química y Biología, Universidad de Santiago de Chile, Alameda, 3363 Santiago, Chile; Laboratorio de Metagenómica Bacteriana, Centro de Biotecnología Acuícola, Departamento de Biología, Facultad de Química y Biología, Universidad de Santiago de Chile, Alameda, 3363 Santiago, Chile; Laboratorio de Virología de Hongos, Departamento de Biología, Facultad de Química y Biología, Universidad de Santiago de Chile, Alameda, 3363 Santiago, Chile; Laboratorio de Microbiología Ambiental y Extremófilos, Departamento de Ciencias Biológicas y Biodiversidad, Universidad de los Lagos, Fuchslocher, 1305 Osorno, Chile; Centro de Investigación Biomédica, Instituto de Ciencias Biomédicas, Facultad de Ciencias de la Salud, Universidad Autónoma de Chile, El Llano Subercaseaux, 2801 Santiago, Chile; Universidad Científica del Sur, Panamericana Sur Km. 19, Lima, Perú

**Keywords:** Violacein, *Janthinobacterium lividum*, Carbon dioxide, Capnophilic, Carbon fixation

## Abstract

*Janthinobacterium lividum* is a Gram-negative bacterium able to produce violacein, a pigment with antimicrobial and antitumor properties. *Janthinobacterium lividum* colonizes the skin of some amphibians and confers protection against fungal pathogens. The mechanisms underlying this association are not well understood. In order to identify the advantages for the bacterium to colonize amphibian skin we sequenced *Janthinobacterium lividum* strain MTR, a strain isolated from Cajón del Maipo, Chile. The strain has capnophilic behavior, with growth favored by high concentrations (5 %) of carbon dioxide. Its genome is 6,535,606 bp in size, with 5,362 coding sequences and a G + C content of 62.37 %. The presence of genes encoding for products that participate in the carbon fixation pathways (dark CAM pathways), and the entire set of genes encoding for the enzymes of the glyoxylate cycle may explain the capnophilic behavior and allow us to propose that the CO_2_ secreted by the skin of amphibians is the signal molecule that guides colonization by *Janthinobacterium lividum.*

**Electronic supplementary material:**

The online version of this article (doi:10.1186/s40793-015-0104-z) contains supplementary material, which is available to authorized users.

## Introduction

*Janthinobacterium lividum* is a betaproteobacterium common in soil and water in cold-temperate regions [1, 2]. This bacterium produces violacein, a compound with antibiotic and antiviral properties that is toxic for eukaryotic cells [3, 4]. Although the biological function of violacein for *Janthinobacterium lividum* remains unknown, it has been proposed that the compound provides protection against bacterial predators [5, 6]. *Janthinobacterium lividum* is also present in the skin microbiome of some amphibians, where its presence and production of violacein confers protection against the fungal pathogen *Batrachochytrium endrobatidis* [7–9]. This bacterial-amphibian relationship has been classified as symbiotic (mutualism) [7, 10, 11], although the benefit for the bacteria has not been clearly established. Carbon dioxide is secreted by amphibian skin alveoli. Although carbon dioxide is a highly abundant, its role as a signaling molecule in bacteria has not been well characterized. *Janthinobacterium lividum* strain MTR is an isolate from our laboratory that grows best in carbon dioxide concentrations higher than 1 % (with an optimum of 5 %). These carbon dioxide concentrations are close to the ranges (1.2–2.5 % CO_2_) observed in amphibian skin alveoli [12]. Because this property has not been described before in other strains of *Janthinobacterium lividum*, we focused on sequencing the genome of this bacterial strain to elucidate the function of the carbon dioxide as a signal molecule.

## Organism information

### Classification and features

*Janthinobacterium lividum* is a rod-shaped, Gram-negative, motile, aerobic bacterium, commonly isolated from of soil and water of cold regions, such as mountains or glaciers. It is a heterotrophic bacterium with a temperature range of growth between 4 and 30 °C, with a optimum growth at around 25 °C [2], suggesting that is a psychrotolerant bacterium. After several day of cultivation on solid nutrient medium, most of the strains form rubbery colonies that are violet colored due to the production of violacein. The MTR strain of *Janthinobacterium lividum* forms violet-colored colonies with a rubber-like aspect that cannot grow at temperatures above 30 °C, with an optimum growth temperature of 25 °C (Table 1). Under aerobic conditions, strain MTR grows as a planctonic culture without violacein production, while in static cultures it forms a thick dark violet biofilm in the air-broth interface. In static cultures, *Janthinobacterium lividum* MTR also shows capnophilic behavior that has not been described before among other members of the *Janthinobacterium* genus. Comparison of bacterium grown on Luria, Todd Hewitt, nutrient, Sabouraud, Mueller Hinton, and MacConkey solid media shows that colonies of *Janthínobacterium lividum* strain MTR grow best on Todd Hewitt, nutrient and MacConkey plates. No growth was observed on Sabouraud plates. Growth was more rapid on plates of nutrient medium, where violacein production was evident at only 24 h of culture at 25 °C, while violacein production was observed on plates of Luria and Mueller Hinton medium at 72 h of growth. In all tested culture media *Janthinobacterium lividum* MTR formed bright and convex colonies in the first 72 h. At 72 h, the colony morphology began to change, becoming dark violet, bright, rugous and raised by seven days of cultivation.

**Table 1:** Classification and general features of *Janthinobacterium lividum* strain MTR^T^ [40]

MIGS ID	Property	Term	Evidence code^a^
	Classification	Domain Bacteria	TAS [41]
		Phylum *Proteobacteria*	TAS [42]
		Class *Betaproteobacteria*	TAS [43, 44]
		Order *Burkholderiales*	TAS [45]
		Family *Oxalobacteraceae*	TAS [1, 43]
		Genus *Janthinobacterium*	TAS [1]
		Species *Janthinobacterium lividum*	TAS [1]
		(Type) strain: *Strain* ^*T*^ *MTR*	
	Gram stain	Negative	TAS [30]
	Cell shape	Rod	TAS [1]
	Motility	Motile	TAS [30]
	Sporulation	Nonsporulated	TAS [1]
	Temperature range	4–30 °C	TAS [46, 47]
	Optimum temperature	25 °C	TAS [46, 47]
	pH range; Optimum	5.0–8.0; 7.0	IDA
	Carbon source	Heterotrophic	TAS [47]
MIGS-6	Habitat	Aquatic and soil	TAS [46]
MIGS-6.3	Salinity	Non reported	NAS
MIGS-22	Oxygen requirement	Aerobic	TAS [30]
MIGS-15	Biotic relationship	Free-living	TAS [47]
MIGS-14	Pathogenicity	Non-pathogen	NAS
MIGS-4	Geographic location	Cajón del Maipo, Santiago Metropolitan Region, Chile	NAS
MIGS-5	Sample collection	2009	IDA
MIGS-4.1	Latitude	−33.56	IDA
MIGS-4.2	Longitude	−70.46	IDA
MIGS-4.4	Altitude	Non reported	NAS

^a^Evidence codes - *IDA* Inferred from Direct Assay, *TAS* Traceable Author Statement (i.e. a direct report exists in the literature), *NAS* Non-traceable Author Statement (i.e., not directly observed for the living, isolated sample, but based on a generally accepted property for the species, or anecdotal evidence). These evidence codes are from the Gene Ontology Project [48]

**Fig. 1:**
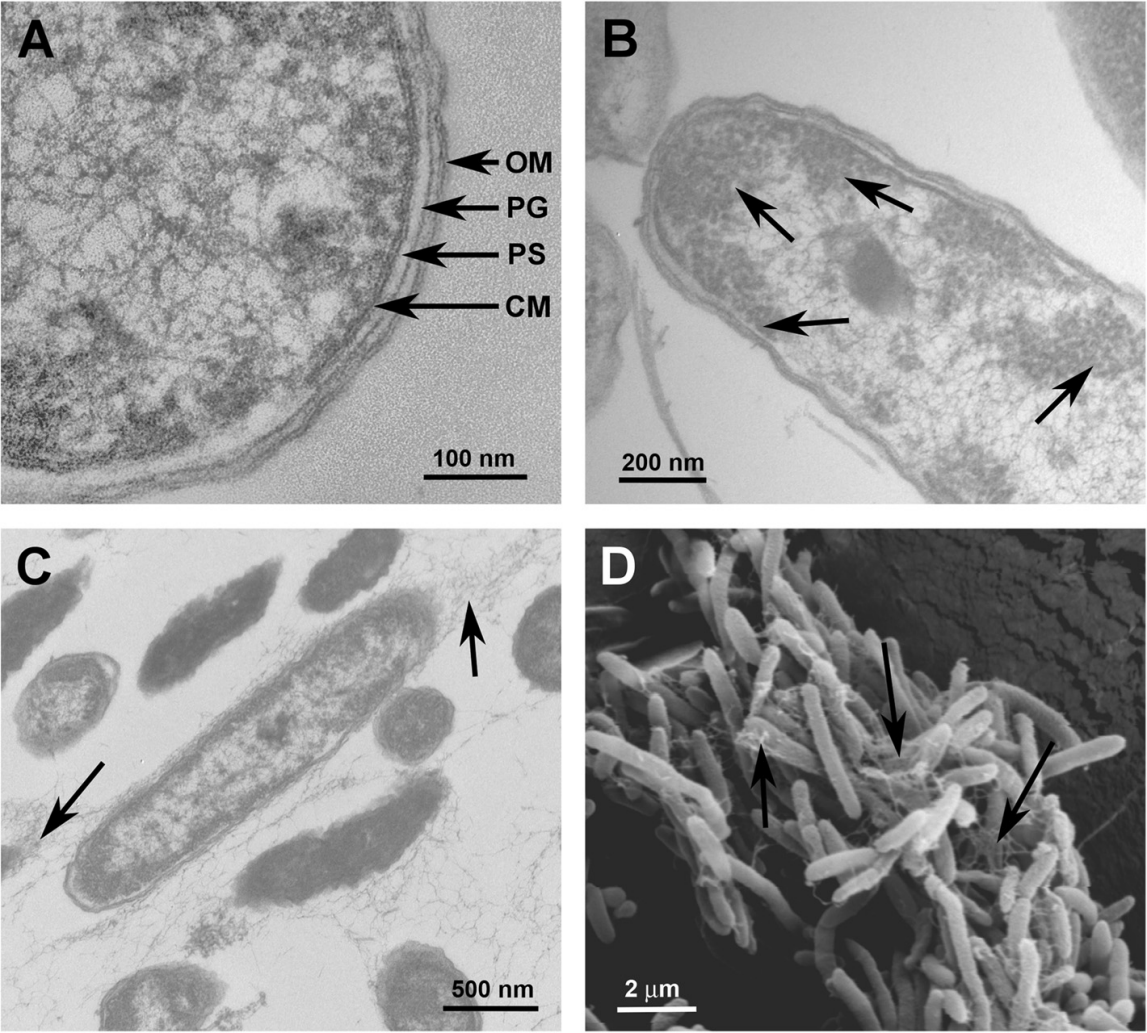
Electron micrographs of the *Janthinobacterium lividum* strain MTR. **a**, **b** and **c**, ultrathin sections visualized by transmission electron microscope. **d**, sample of bacterial cells shading with metallic gold and visualized by scanning electron microscope. The arrows in (**a**) indicate the outer membrane (OM), the peptidoglycan (PG), the periplasmic space (PS) and the cytoplasmic membrane (CM). The arrows in (**b**) show areas of high concentration of ribosomes. The arrows in (**c**) and (**d**) indicate regions where exopolymeric substances can be seen clearly (EPS)

Ultrastructural analysis by electron microscopy of ultrathin sections of *Janthinobacterium lividum* strain MTR confirmed the presence of an exopolymeric substance that would be involved in the formation of biofilms (see arrows in Fig. 1, c and d). High magnification showed the clearly layered structure of the Gram-negative cell envelope or outer membrane, the peptidoglycan cell wall, and the cytoplasmic or inner membrane (see arrows in Fig. 1a). It was also possible to observe high concentrations of ribosomes in the bacterial cytoplasm (see arrows in Fig. 1b), indicating a high level of protein biosynthesis. The bacterial cells are rod-shaped, approximately 2.5 μm long and 0.65 μm in diameter (Fig. 1).

Antibiogram profiles using the Kirby-Bauer method [13] showed that *Janthinobacterium lividum* MTR is resistant to clindamycin, tetracycline, ampicillin, penicillin, cefuroxime, cephaloridine, and piperacillin.

**Fig. 2:**
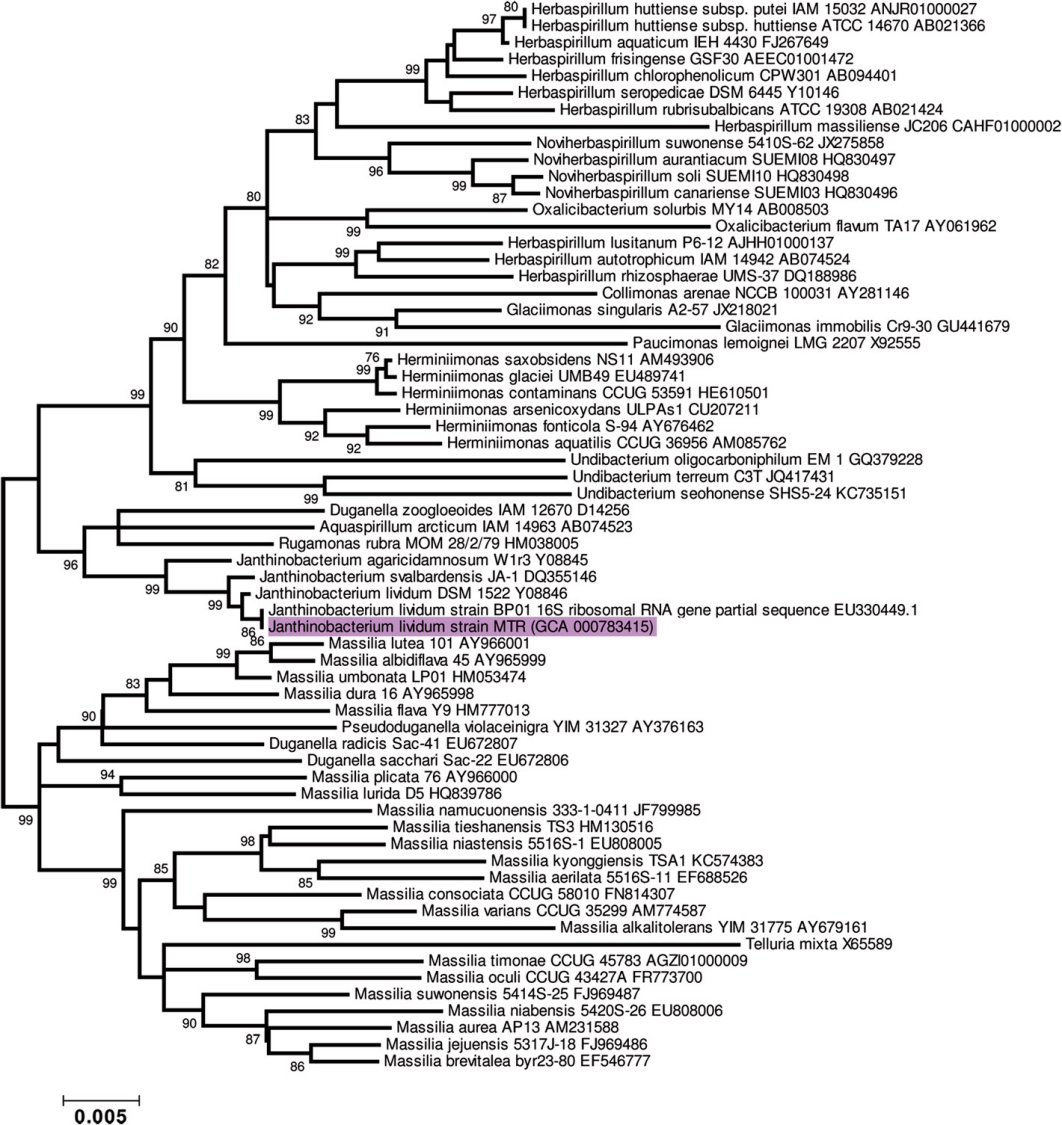
Phylogenetic relationship of the *Janthinobacterium lividum* strain MTR to other members of the *Oxalobacteraceae* family: The evolutionary history was inferred using the neighbor-joining method [49]. The optimal tree with a branch length sum = 0.15329841 is shown. The percentage of replicate trees (higher than 75) in which the associated taxa clustered together in the bootstrap test (1000 replicates) are shown next to the branches [50]. The tree is drawn to scale, with branch lengths in the same units as those of the evolutionary distances used to infer the phylogenetic tree. The evolutionary distances were computed using the maximum composite likelihood method [51] and are in the units of the number of base substitutions per site. The rate variation among sites was modeled with a gamma distribution (shape parameter = 1). The analysis involved 34 nucleotide sequences. All positions containing gaps and missing data were eliminated. There were a total of 1429 positions in the final dataset. Evolutionary analyses were conducted in MEGA5 [52]. The *Janthinobacterium lividum* strain MTR is highlighted in violet

Phylogenetic reconstruction using the sequences of 16S rRNA obtained from the genome sequence of *Janthinobacterium lividum* MTR and EZtaxon database [14] confirmed that our strain belongs to the domain *Bacteria*, the phylum *Proteobacteria*, the class *Betaproteobacteria*, the order *Burkholderiales*, the family *Oxalobacteraceae*, the genus *Janthinobacterium*, and the species *Janthinobacterium lividum*, which is closely related to *Janthinobacterium lividum* BP01 isolated in Alaskan soil (Fig. 2) [15]. The 16S rRNA sequence of the MTR and BP01 strains of *Janthinobacterium lividum* share 100 % identity, although the 16S rRNA sequence of BP01 is shorter than it counterpart in the MTR strain.

*Janthinobacterium lividum* strain MTR was isolated from soil samples (5 g) taken from a forest of native *Drimys winteri* var. *chilensis* A. Gray trees located in the Maipo Valley in central Chile. Samples were dissolved in 40 mL of sterile distilled water and incubated at 40 °C for 1 h to diminish the load of mesophile bacteria. This suspension (100 μL) was mixed with 3 mL of molten soft agar of salt medium (KNO_3_ 2.0 g/L, MgSO_4_ ·7H_2_O 2.0 g/L, K_2_HPO_4_ 2.0 g/L, MgSO_4_·7H_2_O 0.05 g/L, CaCO_3_ 0.02 g/L, FeSO_4_·7H_2_O 0.01 g/L) and poured over salt medium plates supplemented with starch 1 %, casein 0.03 % and cycloheximide (10 μg/mL). After 10 days of culture at 25 °C, blue-violet colonies were streaked over plates of nutritive medium and grown at 25 °C. Each isolate was replated at least 4 times until an axenic culture was obtained. The initial identity of bacterial isolate was determined by 16S rRNA sequencing. Each isolate of *Janthinobacterium lividum* was grown in nutrient media in 2 mL static cultures at 25 °C, using carbon dioxide concentrations of 5 % and 0.03 %. After 48 h, growth was determined by CFU using the microdrop method. *Janthinobacterium lividum* strain MTR showed the best growth rate at 5 % of carbon dioxide and consequently this colony was selected for genomic sequencing.

## Genome sequencing information

### Genome project history

*Janthinobacterium lividum* strain MTR was selected for sequencing because of its capnophilic behavior, with optimum growth at carbon dioxide concentrations close to those estimated to be present on the skin of amphibian due to gas exchange. The genome project and draft genome sequence of the MTR strain of *Janthinobacterium lividum* were deposited at DDBJ/EMBL/GenBank under the accession code of the master record JRRH00000000, nucleotide sequences JRRH01000001-JRRH01000114. Sequencing was performed in March 2014 at the Genomic and Bioinformatic Center of the Universidad Mayor and released on November 14th 2014. Assembly and annotation were done by the bioinformatic support unit at the Universidad de Santiago and were completed in October 2014. The draft genome shows a mean coverage of 30x. Table 2 provides a summary of the project.

**Table 2:** Project information

MIGS ID	Property	Term
MIGS 31	Finishing quality	30 ×
MIGS-28	Libraries used	Paired-end
MIGS 29	Sequencing platforms	Illumina MiSeq
MIGS 31.2	Fold coverage	144 ×
MIGS 30	Assemblers	Velvet v.1.2.10
MIGS 32	Gene calling method	RAST
	Locus tag	NC77
	Genbank ID	JRRH01000001-JRRH01000114
	GenBank date of release	11/14/2014
	GOLD ID	Gp0112111
	BIOPROJECT	PRJNA263254
MIGS 13	Source material identifier	MTR1474
	Project relevance	Environment

### Growth conditions and genomic DNA preparation

*Janthinobacterium lividum* strain MTR was grown in nutritive broth at 25 °C by 48 h. Total DNA was isolated from 3 mL of liquid culture using the MasterPure^TM^ DNA purification kit (Epicentre®). Cells were first collected by centrifugation at 13,000 g for 5 min, resuspended in 300 μL of tissue and cell lysis solution and treated with 100 μg of lyzozyme at 37 °C for an hour. After lyzozyme treatment, cell lysis was achieved by adding 50 μg of proteinase K and incubation at 37 °C for an hour with brief agitation every 5 min. After lysis, RNA was removed by treatment with 10 μg of RNAse A for 5 min at 37 °C. Protein and cell debris was removed by adding 175 μL of MPC solution, and further centrifugation at 13,000 g for 10 min at 4 °C. The DNA was precipitated by adding 300 μL of isopropanol per each 400 μL of supernatant and was collected by centrifugation at 13000 g for 10 min at 4 °C. The pellet containing DNA was washed with ethanol 70 %, dried using a SpeedVac and resuspended in 50 μL of miliQ water. The DNA was stored at −20 °C until its subsequent use.

### Genome sequencing and assembly

The genome of the *Janthinobacterium lividum* strain MTR was sequenced using Illumina MiSeq, a sequencing-by-synthesis technology, which generates 2 × 250 reads (paired-ends). The reads were analyzed by FastQC v.0.10.1 [16] and low-quality sequences were removed by Trimmomatic v.0.32 before assembly. The trimmed sequences were assembled *de novo* using an assembling coverage of 120x, with the Velvet Assembler program (v1.2.10 p).

### Genome annotation

Annotations was performed using RAST [17], identifying a total of 5,774 protein encoding genes. The tRNAs were identified by tRNAscan-SE v.1.23 [18] and the rRNAs by *RNAmmer**v.*1.2Server [19]. The proteins identified by RAST were used to determine clusters of orthologous groups with the WebMGA server [20], CRISPRs were evaluated by CRISPRfinder [21], transmembrane helix domains were determined by TMHMM 2.0c [22] and signal peptides were estimated by SignalP v.4.0 [23].

## Genome properties

**Fig. 3:**
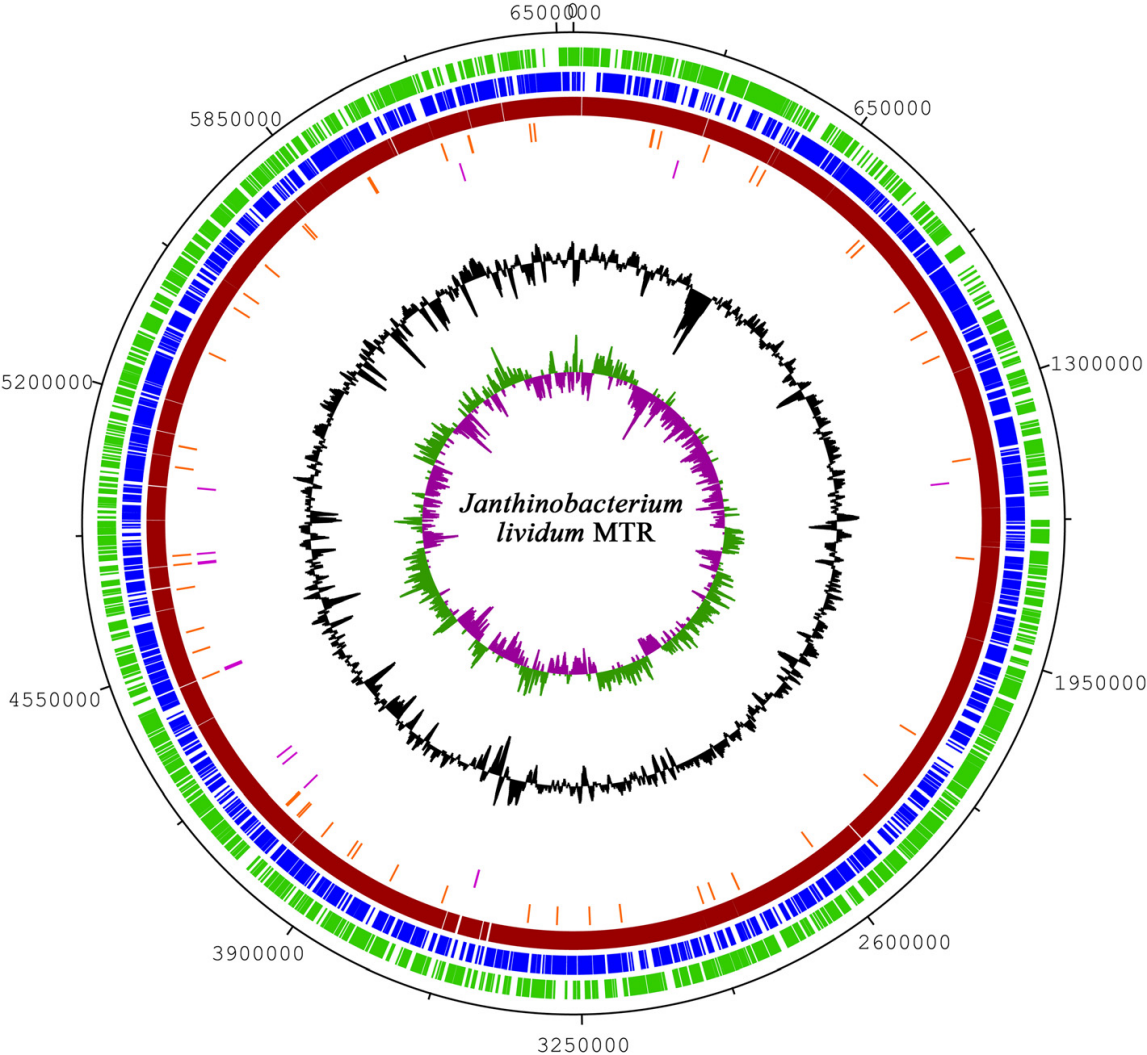
Circular representation of *Janthinobacterium lividum* strain MTR genome: The figure shows the forward CDS (green), reverse CDS (blue), total CDS, coding genome (red), tRNAs (orange), rRNAs (violet), GC content (black), and GC skew (purple/olive)

The draft genome properties and statistics of *Janthinobacterium lividum* strain MTR (Accession CP006828) are shown in Tables 3 and 4. The assembly the MTR strain resulted in 114 contigs, with sizes ranging from 209 to 472,391 bp (N50, 154,343 bp). The total draft genome had a length of 6,535,606 bp, with a G + C content of 62.37 %. The draft genome was shown to encode 5,876 putative genes, 5,362 protein-coding genes, 408 pseudogenes, and 106 genes for RNAs (87 genes encoding for tRNA, two complete 5S-16S-23S operons) (Fig. 3). Approximately 67.69 % of the genes encode for putative proteins with known functions (Table 3). Table 4 presents the distribution of genes in functional COG categories.

**Table 3:** Genome statistics

Attribute	Value	% of total
Genome size (bp)	6,535,606	100.00
DNA coding (bp)	5,330,283	81.56
DNA G + C (bp)	4,076,257	62.37
DNA scaffolds	114	100.00
Total genes	5,876	100.00
Protein coding genes	5,362	91.25
RNA genes	106	1.80
Pseudo-genes	408	6.94
Genes in internal clusters	2,639	44.91
Genes with predicted functions	3,978	67.69
Genes assigned to COGs	4,181	79.94
Genes with Pfam domains	4,268	79.59
Genes with signal peptides	1,250	21.27
Genes with transmembrane helices	1,065	18.12
CRISPR repeats	0	0.00

**Table 4:** Number of genes associated with general functional COG categories

Code	Value	% age	Description
J	183	3.41	Translation, ribosomal structure and biogenesis
A	3	0.06	RNA processing and modification
K	474	8.84	Transcription
L	148	2.76	Replication, recombination and repair
B	3	0.06	Chromatin structure and dynamics
D	39	0.73	Cell cycle control, Cell division, chromosome partitioning
V	82	1.53	Defense mechanisms
T	483	9.01	Signal transduction mechanisms
M	272	5.07	Cell wall/membrane biogenesis
N	190	3.54	Cell motility
U	178	3.32	Intracellular trafficking and secretion
O	181	3.38	Posttranslational modification, protein turnover, chaperones
C	273	5.09	Energy production and conversion
G	302	5.63	Carbohydrate transport and metabolism
E	374	6.98	Amino acid transport and metabolism
F	85	1.59	Nucleotide transport and metabolism
H	179	3.34	Coenzyme transport and metabolism
I	172	3.21	Lipid transport and metabolism
P	291	5.43	Inorganic ion transport and metabolism
Q	98	1.83	Secondary metabolite biosynthesis, transport and catabolism
R	534	9.96	General function prediction only
S	441	8.22	Function unknown
-	1,181	22.03	Not in COGs

The total is based on the total number of protein coding genes in the genome

## Insights from the genome sequence

Currently, the genomes of eight strains of *Janthinobacterium* spp. have been deposited in the GenBank database. The strains *Janthinobacterium* sp. CG3, *Janthinobacterium* sp. HH01, *Janthinobacterium* sp. Marseille, *Janthinobacterium* sp. RA13, *Janthinobacterium agaricidamnosum*NBRC102515, *Janthinobacterium lividum* PAMC25724, *Janthinobacterium lividum* RIT308 and *Janthinobacterium lividum* MTR have been sequenced to different levels. A complete genome has only been reported for *Janthinobacterium* sp. Marseille and *Janthinobacterium agaricidamnosum*NBRC102515. Although all these bacteria have been classified as belonging to the *Janthinobacterium* genus, detailed analysis of the 16S rRNA sequence using EZTaxon showed that *Janthinobacterium* sp. HH01 and *Janthinobacterium* sp. Marseille are closer to others members of the *Oxalobactericeae* family, *Duganella phyllosphaerae* and *Herminiimonas glaciei*, respectively. The 16S rRNA of *Janthinobacterium* sp. CG3 showed 98.43 % identity with 16S rRNA of *Janthinobacterium**svalbardensis*, while the 16S rRNA of *Janthinobacterium* sp. RA13 had 100 % identity with the 16S rRNA of *Janthinobacterium lividum*DSM1522. This analysis also indicated that *Janthinobacterium lividum* strain MTR has a 99.86 % of identity with the 16S rRNA of *Janthinobacterium lividum*DSM1522. This suggest that there are only four genomes of the bonafide species *Janthinobacterium* in Genbank. Comparisons of these genomes shows that *Janthinobacterium lividum* strain MTR has a similar GC percentage (62.4 %) to those of the strains RIT308 and RA13, and higher than that of the strain PAMC25724. *Janthinobacterium lividum* strain MTR has the largest genome, with 6.5 Mb, followed by the strains RA13 (6.4 Mb), RIT308 (6.2 Mb) and PAMC25724 (4.98 Mb). Although *Janthinobacterium lividum* strain MTR has the largest genome among lividum strains, it encodes for only 5362 proteins, fewer than the 5712 and 5431 putative proteins encoded in RA13 and RIT308 strains, respectively. Comparison of the proteins encoded in *Janthinobacterium lividum* strain MTR shows that our strain is most closely related to *Janthinobacterium lividum* RIT308. Approximately 45.76 % of the putative proteins encoded in *Janthinobacterium lividum* MTR have their closest homologues in *Janthinobacterium lividum* RIT308, while 42.9 % and 4.70 % of genes have their closest homologues in *Janthinobacterium* sp RA13 and *Janthinobacterium lividum* PAMC25724, respectively. In relation to strain specific genes, 12 % of putative protein encoded by our strain share less than 80 % of identity with proteins encoded in the genome of others members of the lividum species. Whole genome comparison with blastN showed similar results. *Janthinobacterium agaricidamnosum*, *Janthinobacterium* sp. GC3, *Janthinobacterium lividum* RIT308, *Janthinobacterium* sp. RA13, and *Janthinobacterium lividum* PAMC25724 form a clade in which *Janthinobacterium lividum* MTR is closer to the bacterial strains *Janthinobacterium* sp. RA13 and *Janthinobacterium lividum* RIT308. As was predicted by the 16S rRNA sequence analysis, whole genome comparison revealed that *Janthinobacterium* HH01 is closer to a member of *Duganella* while *Janthinobacterium* sp. Marseille is closer to a member of the *Herminiimonas* group (Additional file 1: Figure S1). Comparison using ANI (Average Nucleotide Index) indicated that *Janthinobacterium lividum* strain MTR share a 92.68 % and 92.38 % with RA13 and RIT308 strains, respectively. These values are below of the threshold percentage (95 %) to be considered members of the same species (Additional file 2: Table S2). Interesting, among the genomes analyzed, ANI values higher than 94 % were not observed. These results suggest that classification inside of *Janthinobacterium* genus must be carefully analyzed and reconsidered in future works.

Resistance to penicillin, first- and second-generation carbapenems and cephalosporins, may be due to the presence of a gene homologous to THIN-B (KHA75569.1), which is a broad spectrum β-lactamase [24]. Resistance to tetracycline may be due to the high number of efflux pumps identified in the genome (KHA80475.1, KHA80070.1, KHA80071.1, KHA79872.1, KHA78654.1, KHA78419.1, KHA78456.1, KHA77902.1, KHA76948.1, KHA76949.1, EZP37289.1, KHA75757.1) [25].

### Extended insights

Comparison of metabolic characteristics deduced from COGs to those of other sequenced species of *Janthinobacterium* showed that *Janthinobacterium lividum* strain MTR has more genes in the categories C (energy production and conversion) and P (inorganic ion transport and metabolisms). With 279 genes in category C, our strain has the largest number of genes related to electron transport (citocromes, plastocianin, etc.) and enzymes with putative coenzyme F420-dependent N5,N10-methylene tetrahydromethanopterin reductase activity. These enzymes are present in methanogenic microorganisms and reduce CO_2_ to methane [26]. The large number of genes related to this function may explain the high growth rate of this strain observed in CO_2_ rich atmospheres, in which CO_2_ could serve as an alternative electron acceptor. In agreement with this observation, our strain has a large number of genes in category P encoding for ABC-type nitrate/sulfonate/bicarbonate transport systems and ferric uptake, suggesting that this strain has high requirements of CO_2_ and electron transport.

The reconstruction of metabolic pathways using KEGG showed the presence of enzymes that fix CO_2_ in C4 plants in dark CAM (Crassulacean acid metabolism). This pathway fixes CO_2_ in two steps, the first of which is catalyzed by phosphoenolpyruvate carboxylase and fixes CO_2_ through the formation of oxaloacetate from PEP and CO_2_. The second step is catalyzed by malate dehydrogenase and transforms oxaloacetate into malate [27]. In *Janthinobacterium lividum* strain MTR, this pathway may be used as an anaplerotic reaction to restore or increase the molecules that make up the TCA cycle, which improves metabolism under aerobic condition. Another possible role of the CAM pathway is that excess malate is used to produce piruvate, the molecule that initiates gluconeogenesis. *Janthinobacterium lividum* strain MTR encodes for a decarboxylating malate dehydrogenase (E1.1.1.39) that converts malate into pyruvate [28]. However, in this step, one carbon atom is lost as CO_2_ so that a positive carbon balance is not expected. Interestingly, our strain also encodes for the enzymes that make up the glyoxylate cycle, which facilitates the conversion of acetyl-CoA into malate and allows for avoidance of the decarboxylation steps of the TCA cycle. Malate in turn converts into pyruvate, which initiates gluconeogenesis [29]. These steps allow for retaining three quarters of the carbon atoms. Because the CAM pathway fixes one carbon atom, its coupling with the glyoxylate cycle should increase the fraction of carbon retained in gluconeogenesis. In the stationary phase, *Janthinobacterium lividum* forms a strong biofilm that is rich in exopolysaccharides [30]. The increase in TCA and gluconeogenesis metabolism by the combination of the CAM pathway, and glyoxylate cycle may improve the production of exopolysaccharide in the stationary phase in which complex carbon sources are not available. In fact, our strain contains all the enzymes required for glycolysis and gluconeogenesis so that it is highly plausible that our bacterium combines these pathways. Interestingly, the glyoxylate cycle is a virulence factor in some pathogenic microorganisms. Inhibition or deletion of isocitrate lyase, the key enzyme of glyoxylate cycle, reduces the survival of fungi and mycobacteria in hosts, and has been the target for designing novel antimicrobial agents [31–34].

Although capnophilic behavior is present in several bacteria, among them human pathogens such as *Helicobacter**pilory*, *Campylobacter jejuni* subsp jejuni and *Aggregatibacter* spp, the exact role of CO_2_ requirement is not well understand [35–37]. Several capnophilic bacteria are microaerophilic and do not grow well in high concentrations of O_2,_ so that O_2_ displacement by CO_2_ may explain this characteristic. Interestingly *Mannheimia**succiniciproducens*, a heterotrophic bacterium incapable of aerobic respiration, requires CO_2_ to produce fumarate from PEP. *Mannheimia**succiniciproducens* lacks of some enzymes required for oxidative phosphorylation so that electron flux is maintained using fumarate as electron acceptor [38, 39]. In *Campylobacter**jujuni* and *Helicobacter**pilori*, the presence of PFOR:FldA:FqrB complexes generates pyruvate via CO_2_ fixation, which may explain the capnophilic behavior of the two bacteria. Since *Janthinobacterium lividum* strain MTR has the entire set of enzymes required for phosphorylative oxidation using O_2_ as an electron acceptor and does not encode a pyruvate ferredoxin oxidoreductase, our data suggest that *J. lividum* strain MTR and others species of *Janthinobacterium* are capnophilic due to different mechanisms, in which CO_2_ improves the metabolic rate of TCA and gluconeogenesis.

## Conclusions

The genome sequence of *Janthinobacterium lividum* strain MTR revealed the presence of enzymes that allow carbon fixation (dark CAM pathway). These enzymes, in combination with the glyoxylate cycle, may increase the efficiency of gluconeogenesis by using intermediaries from the TCA cycle, which would explain capnophilic behavior. To our knowledge, this mechanism has not been proposed before and could be a strategy to improve growth in regions with high amounts of O_2_ and CO_2_, such as the skin of amphibians. Detailed comparison should be made of the *Janthinobacterium lividum* strain MTR genome to those of other sequenced strains of *Janthinobacterium lividum* or other capnophilic bacteria to determine if this proposed mechanism is common among these bacteria.

## Additional files

Additional file 1: Figure S1. Whole genome comparison among bacteria from *Janthinobacterium* genus. The figure shows the whole genome comparison of bacteria belonging to the genuses *Janthinobacterium*, *Duganella* and *Herminiimonas*. Comparison was performed with Gegenees software (PLoS One. 2012;7(6):e39107. doi:10.1371/journal.pone.0039107). Parameters were fixed as follow: fragments = 200 bp, overlapping regions = 100 pb. Genome comparisons were done with blastN algorithm. Panel A shows the average normalized BLAST scores for all fragments in each genome (compared against other genomes). Panel B shows a phylogenomic reconstruction inferred with the Neighbor-joining algorithm (by considering the distance matrix generated by Gegenees software). Reconstruction was done with Mega 6.0 software. *Janthinobacterium lividum* MTR is highlighted with a solid red rhomboid. (DOC 590 kb) Additional file 2: Table S1. The table shows the ANI values among the strain analyzed. ANI values were calculated according to Goris *et al.* 2007 (Goris J., Konstantinidis K., Klappenbach J., Coenye T., Vandamme P., Tiedje J. DNA-DNA hybridization values and their relationship to whole-genome sequence similarities. *Int J Syst Evol Microbiol.* 2007 57(Pt 1):81–91), using web application (http://enve-omics.ce.gatech.edu/ani/) (Figueras M., Beaz-Hidalgo R., Hossain M., Liles M. Taxonomic affiliation of new genomes should be verified using average nucleotide identity and multilocus phylogenetic analysis. *Genome Announc*. 2014 2(6). pii: e00927-14. doi:10.1128/genomeA.00927-14). (DOC 235 kb)

## Abbreviations

TCA: Tricarboxylic Acids
PEP: Phosphoenolpiruvate
CAM: Crassulacean acid metabolism
