# Supplementary material for: Draft genome sequence of Janthinobacterium lividum strain MTR reveals its mechanism of capnophilic behavior
Source: Stand Genomic Sci. 2015 Nov 24;10:110. doi: 10.1186/s40793-015-0104-z (PMC4657372; doi:10.1186/s40793-015-0104-z)
Supplement: Additional file 1: Figure S1. — Whole genome comparison among bacteria from Janthinobacterium genus. The figure shows the whole genome comparison of bacteria belonging to the genuses Janthinobacterium, Duganella and Herminiimonas. Comparison was performed with Gegenees software (PLoS One. 2012;7(6):e39107. doi:10.1371/journal.pone.0039107). Parameters were fixed as follow: fragments = 200 bp, overlapping regions = 100 pb. Genome comparisons were done with blastN algorithm. Panel A shows the average normalized BLAST scores for all fragments in each genome (compared against other genomes). Panel B shows a phylogenomic reconstruction inferred with the Neighbor-joining algorithm (by considering the distance matrix generated by Gegenees software). Reconstruction was done with Mega 6.0 software. Janthinobacterium lividum MTR is highlighted with a solid red rhomboid. (DOC 590 kb) [file 40793_2015_104_MOESM1_ESM.doc]

A


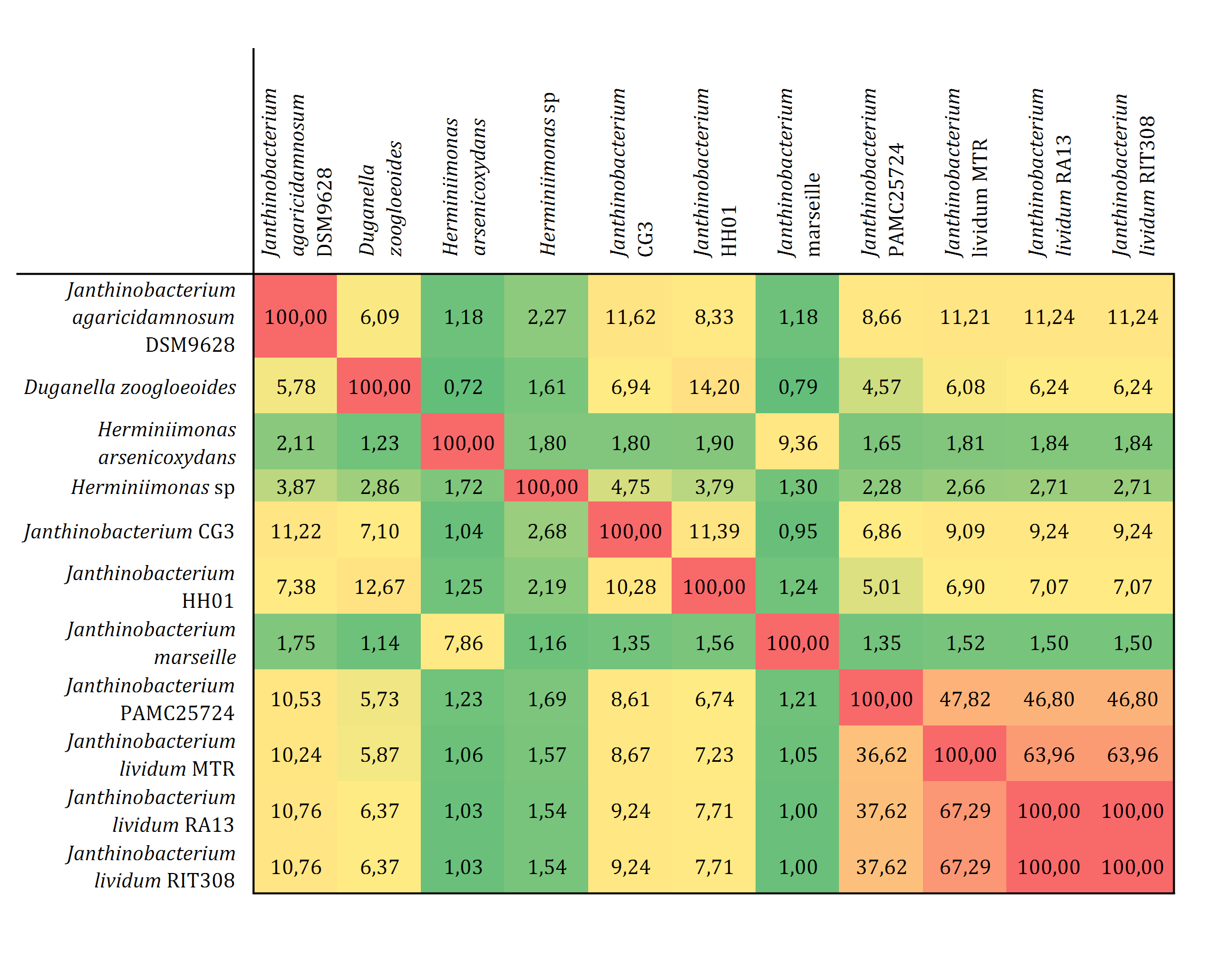


**B**

**Additional file 1: Figure S1: Whole genome comparison among bacteria from *Janthinobacterium* genus**. The figure shows the whole genome comparison of bacteria belonging to the genuses *Janthinobacterium*, *Duganella* and *Herminiimonas*. Comparison was performed with Gegenees software (PLoS One. 2012;7(6):e39107. doi: 10.1371/journal.pone.0039107). Parameters were fixed as follow: fragments=200 bp, overlapping regions=100 pb. Genome comparisons were done with blastN algorithm. Panel A shows the average normalized BLAST scores for all fragments in each genome (compared against other genomes). Panel B shows a phylogenomic reconstruction inferred with the Neighbor-joining algorithm (by considering the distance matrix generated by Gegenees software). Reconstruction was done with Mega 6.0 software. *Janthinobacterium lividum* MTR is highlighted with a solid red rhomboid.
