# Supplementary material for: Draft genome sequence of Janthinobacterium lividum strain MTR reveals its mechanism of capnophilic behavior
Source: Stand Genomic Sci. 2015 Nov 24;10:110. doi: 10.1186/s40793-015-0104-z (PMC4657372; doi:10.1186/s40793-015-0104-z)
Supplement: Additional file 2: Table S1. — The table shows the ANI values among the strain analyzed. ANI values were calculated according to Goris et al. 2007 (Goris J., Konstantinidis K., Klappenbach J., Coenye T., Vandamme P., Tiedje J. DNA-DNA hybridization values and their relationship to whole-genome sequence similarities. Int J Syst Evol Microbiol. 2007 57(Pt 1):81–91), using web application (http://enve-omics.ce.gatech.edu/ani/) (Figueras M., Beaz-Hidalgo R., Hossain M., Liles M. Taxonomic affiliation of new genomes should be verified using average nucleotide identity and multilocus phylogenetic analysis. Genome Announc. 2014 2(6). pii: e00927-14. doi:10.1128/genomeA.00927-14). (DOC 235 kb) [file 40793_2015_104_MOESM2_ESM.doc]

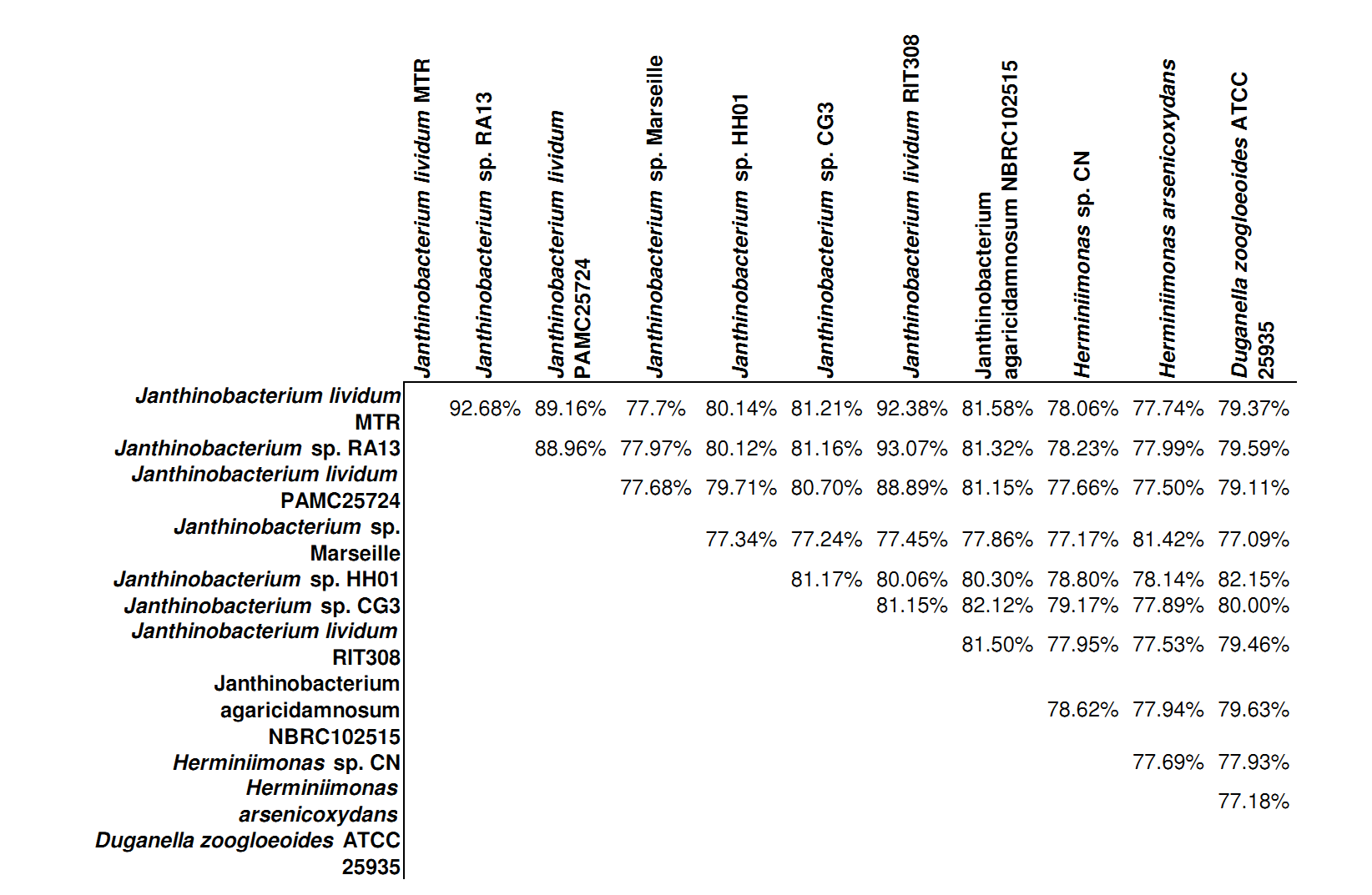


**Additional file 2: Table S1**: The table shows the ANI values among the strain analyzed. ANI values were calculated according to Goris *et al* 2007 (Goris J., Konstantinidis K., Klappenbach J., Coenye T., Vandamme P., Tiedje J. **DNA-DNA hybridization values and their relationship to whole-genome sequence similarities.** *Int J Syst Evol Microbiol.* 2007 **57**(Pt 1):81-91), using web application (<http://enve-omics.ce.gatech.edu/ani/>) (Figueras M., Beaz-Hidalgo R., Hossain M., Liles M. **Taxonomic affiliation of new genomes should be verified using average nucleotide identity and multilocus phylogenetic analysis**. *Genome Announc*. 2014 **2**(6). pii: e00927-14. doi: 10.1128/genomeA.00927-14)
